# Expression of therapy-induced senescence markers in breast cancer samples upon incomplete response to neoadjuvant chemotherapy

**DOI:** 10.1042/BSR20210079

**Published:** 2021-05-20

**Authors:** Tareq Saleh, Ahmad Alhesa, Mahmoud Al-Balas, Omar Abuelaish, Ahmad Mansour, Heyam Awad, Mohammed El-Sadoni, Valerie J. Carpenter, Bilal Azab

**Affiliations:** 1 Department of Basic Medical Sciences, Faculty of Medicine, The Hashemite University, Zarqa 13133, Jordan; 2 Department of Pathology, Microbiology, and Forensic Medicine, School of Medicine, The University of Jordan, Amman 11942, Jordan; 3 Department of General and Special Surgery, Faculty of Medicine, The Hashemite University, Zarqa 13133, Jordan; 4 Department of General Surgery, Royal Medical Services, Amman, Jordan; 5 Department of Pathology and Laboratory Medicine, University of Cincinnati College of Medicine, Cincinnati, OH 45219, U.S.A.; 6 Department of Pharmacology and Toxicology, School of Medicine, Virginia Commonwealth University, Richmond, VA 23298, U.S.A.

**Keywords:** breast cancers, H3K9Me3, Lamin B1, Neoadjuvant Chemotherapy, p21CIP1, Senescence

## Abstract

Senescence is a cell stress response induced by replicative, oxidative, oncogenic, and genotoxic stresses. Tumor cells undergo senescence in response to several cancer therapeutics* in vitro* (Therapy-Induced Senescence, TIS), including agents utilized as neoadjuvant chemotherapy (NAC) in the treatment of invasive breast cancer. TIS has been proposed to contribute to adverse therapy outcomes including relapse. However, there is limited evidence on the induction of senescence in response to NAC in clinical cancer and its contribution to disease outcomes. In this work, the expression of three senescence-associated markers (p21^CIP1^, H3K9Me3 (histone H3 lysine 9 trimethylation), and Lamin B1) was investigated in breast cancer samples that developed partial or incomplete pathological response to NAC (*n*=37). Accordingly, 40.54% of all samples showed marker expression consistent with a senescence-like phenotype, while the remainders were either negative or inconclusive for senescence (2.70 and 56.8%, respectively). Moreover, analysis of core-needle biopsies revealed minimal changes in p21^CIP1^ and H3K9Me3, but significant changes in Lamin B1 expression levels following NAC, highlighting a more predictive role of Lamin B1 in senescence detection. However, our analysis did not establish an association between TIS and cancer relapse as only three patients (8.1%) with a senescence-like profile developed short-term recurrent disease. Our analysis indicates that identification of TIS in tumor samples requires large-scale transcriptomic and protein marker analyses and extended clinical follow-up. Better understanding of *in vivo* senescence should elucidate its contribution to therapy outcomes and pave the way for the utilization of senolytic approaches as potential adjuvant cancer therapy.

## Introduction

Breast cancer is the most commonly diagnosed female malignancy in the United States [1], and the second leading cause of cancer-related deaths in women worldwide [2]. Since the standard-of-care for invasive breast cancer patients has improved dramatically, the number of breast cancer survivors has increased; however, this increase in the surviving population is associated with increasing rates of cancer recurrence [3]. For example, in Dutch women, the regional recurrence and distant metastasis rates over the first 10 years post-therapy ranged from 7.5 to 26% with luminal invasive carcinoma [4], while, in a population of breast cancer female patients in the United Kingdom, the 20-year recurrence rate was 10–17%, depending on the biological subtype and stage [5]. In parallel, there is increased utilization of neoadjuvant chemotherapy (NAC) for the treatment of breast cancer as approximately 17–79% of patients receive a regimen of NAC depending on their biologic subtype [6,7]. Interestingly, the recurrence rate is higher in certain breast cancer patients receiving NAC (mainly, anthracycline-based therapy) in contrast with patients that did not receive the same treatment, strongly suggesting the possible contribution of cellular responses to chemotherapy as mechanisms of cancer recurrence [8]. Thus, there is an avid need for the identification of novel mechanisms that might contribute to disease recurrence, especially in cancers that develop partial response to therapy; a better understanding of these mechanisms should pave the way for a more effective anticancer therapy and higher survival rates.

Cellular senescence is a cell state characterized by a stable growth arrest accompanied by transcriptomic and epigenetic alterations [9,10], macromolecular and metabolic changes [11,12], increased lysosomal biogenesis (senescence-associated β galactosidase, SA-β-gal) [13,14], as well as the secretion of a spectrum of chemokines and cytokines collectively named the Senescence-Associated Secretory Phenotype (SASP) [15]. Senescence is an established cell stress response to DNA damaging, targeted and hormonal cancer therapies, hence the name Therapy-Induced Senescence (TIS) [16]. While TIS has been extensively established in many preclinical models, the induction and potential role of senescence in tumor cells of cancer patients receiving anticancer treatment is still under investigation. This is largely because the canonical marker of senescence, SA-β-gal, is difficult to detect in fixed tumor samples and typically requires flash frozen, unfixed samples. For example, the seminal report by Poele et al. showed that senescence is detected in nearly 40% of tumor samples collected from 36 patients with breast cancer receiving a single regimen of NAC (namely, cyclophosphamide, doxorubicin, and 5-fluorouracil) utilizing archived frozen tumor samples (rather than flash frozen, fresh samples) [17]. Another report by Roberson et al. showed evidence of senescence induction based on SA-β-gal staining in freshly frozen tumor samples derived from three lung cancer patients receiving platinum-based/taxane therapy [18]. While the detection of the SA-β-gal staining is a classical approach to identify senescent cells, its sole use as proof of senescence induction has been received with multiple concerns, including non-specificity [19]. Furthermore, it is challenging to obtain fresh human samples to carry out histochemical staining for SA-β-gal on a routine basis. Moreover, while suggestive evidence on senescence induction in cancer patients is provided in the literature, the contribution of TIS to the overall therapeutic outcome is yet to be determined, especially that senescence has been proposed as a mechanism of tumor dormancy and cancer recurrence [20].

In this work, we hypothesized that TIS might be a component of incomplete disease response to NAC, in that, tumor cells may respond to therapy primarily by senescence rather than cell death, which more classically reflects complete pathological response (pCR). Further, we aimed to establish whether the assessment of multiple markers other than SA-β-gal might prove useful for determining senescence in archived tumor samples. The stability of the senescent growth arrest is enforced by the activation of several cell cycle-regulating proteins [21], including cyclin-dependent kinase inhibitors (CDKIs) such as p21^CIP1^ or p16^INK4a^ [22,23]. p21^CIP1^ mediates the growth arrest occurring in replicative exhaustion-induced senescence [24]. Moreover, several studies have established the relationship between p21^CIP1^ and senescence in tumor cells, as increased expression of p21^CIP1^ was shown to be sufficient to drive tumor cells into a stable growth arrest [25]. Importantly, many studies have suggested that p21^CIP1^ is key in the induction of senescence after exposure to anticancer agents, i.e. TIS [26], and in consequence, p21^CIP1^ is a frequently used senescence marker in preclinical models [27]. In addition to the cell cycle regulators, senescent cells undergo structural changes such as nuclear envelop remodeling marked by degradation of nuclear laminar proteins such as Lamin B1; thus, Lamin B1 loss is an established biomarker of senescence [28]. Also, senescent cells develop epigenetic signatures collectively called the Senescence-associated Heterochromatic Foci (SAHF) that contribute to the regulation of expression of proliferation-associated genes [29]. SAHF can include several epigenetic signatures such as histone H3 lysine 9 trimethylation (H3K9Me3) and the Heterochromatin Protein 1 (HP1) [30]. SAHF represent areas of transcriptionally silent and compacted chromatin that result from the presence of repressive H3K9Me3 and absence of activating H3K4Me3 [31]. Thus, the increased expression level of H3K9Me3 has been considered as an indicator for the occurrence of senescence. These features are manifested variably in therapy-induced senescent tumor cells in preclinical models [32]. Therefore, we examined the expression of these three proteins that reflect the previous senescence hallmarks simultaneously. Here, we provide evidence on the expression of the three TIS-associated markers, namely p21^CIP1^, H3K9Me3 and Lamin B1, in breast tumor samples following NAC and developed partial or incomplete response to therapy. Lastly, we include a correlative analysis of the expression of TIS-associated markers and evidence of cancer recurrence in the studied population.

## Materials and methods

### Samples

All samples were obtained from patients diagnosed with non-metastatic, invasive breast carcinoma and between the years 2017 and 2019 with no history or presence of another concomitant malignant neoplasm from the Department of General Surgery/Breast Surgery and Reconstruction in Jordanian Royal Medical Services (JRMS) and the Department of Surgery in Prince Hamza Hospital (PHH), Amman, Jordan. The criteria for inclusion for biochemical analysis were: (*i*) age between 18 and 90 years; (*ii*) diagnosis of one of the following breast cancer subtypes: Invasive Ductal Carcinoma (IDC): IDC Type: Tubular Carcinoma of the Breast; IDC Type: Medullary Carcinoma of the Breast; IDC Type: Mucinous Carcinoma of the Breast; IDC Type: Papillary Carcinoma of the Breast; IDC Type: Cribriform Carcinoma of the Breast and Invasive Lobular Carcinoma (ILC), as diagnosed by the pathology laboratories of JRMS and PHH and patients who received one of the following NAC prior to surgery: docetaxel, adriamycin and cyclophosphamide (TAC), paclitaxel and doxorubicin plus cyclophosphamide (ACP), Adriamycin plus cyclophosphamide (AC), docetaxel plus cyclophosphamide (TC), 5-fluorouracil, epirubicin and cyclophosphamide (FEC) or 5-fluorouracil, epirubicin, cyclophosphamide followed by docetaxel (FEC+D). The criteria for exclusion for the histopathological/biochemical analysis were: (*i*) patients with inoperable, metastatic disease; (*ii*) patients who were deceased throughout the treatment period and were no longer followed up in the oncology clinic; (*iii*) patients who also received radiation or hormonal neoadjuvant therapy (e.g., tamoxifen); (*iv*) patients who had complete response to NAC determined by surgical and radiological assessment.

In this work, our sample included a total of 89 patients diagnosed with a subtype of breast cancer and treatment of the indicated NAC regimens with variable pathological responses (*n*=89). Of those, ten patients were deceased while receiving treatment and were excluded from the histopathological/biochemical analysis. Furthermore, a total of 37 patients who had a partial or no response to NAC as determined by intraoperative staging were only considered for immunohistochemical staining (*n*=37). Formalin-fixed paraffin-embedded (FFPE) breast tumor blocks for all included patients were collected (*n*=37). The diagnosis of a subtype of invasive breast carcinoma has been confirmed histopathologically using Hematoxylin- and Eosin-stained sections of mastectomy specimens following the administration of NAC by three specialized pathologists at JRMS, PHH, and Jordan University Hospital (JUH). All patients underwent Modified Radical Mastectomy (MRM) within a period of 20–30 days following the completion of the last cycle of NAC, which represents the timepoint of sample collection after exposure to chemotherapy. Lastly, core-needle biopsy samples prior to receiving NAC of 11 out of 37 patients were available and collected for further histopathological analysis/biochemical staining (*n*=11).

### Immunohistochemistry

Immunohistochemical staining was performed in 37 resected tumor specimens of patients receiving NAC and 11 core needle biopsy FFPE samples to assess the protein expression of three senescence-associated markers: Lamin B1, H3K9Me3, and p21^CIP1^ as follows. Five-micrometer-thick tissue sections were cut, using a microtome (LEICA RM2125RT) and placed on clean, charged glass slides. The tissue sections were left to dry at 70°C for 20 min. Sections were dewaxed in two changes of xylene for 5 min, rehydrated in ethanol (100%) for 1 min, then immersed in ethanol (95%) for 1 min, and washed twice with distilled water for 5 min to remove residual alcohol. Antigen retrieval was performed by placing the container of the slides in 10 mM sodium citrate buffer solution, pH 6, for 1 h at 95°C in the water bath. The slides were then cooled at room temperature for 30 min.

The slides were washed with phosphate buffered saline (PBS) (pH 7.3 ± 0.10 diluted to 1 l; 64123666, Bio-Rad) for 5 min and then treated with 3% hydrogen peroxide (DQ400-60KE, BioGenex) for 10 min. After that, sections were washed in PBS and treated with 0.1% Triton X-100 in PBS to permeabilize cell membrane/nuclear envelope for 15 min. The slides were washed in PBS and then incubated for 15 min with power block reagent (Catalog Number: DQ400-60KE, BioGenex). The slides were then incubated with mouse monoclonal antibody specific for human Lamin B1 and mouse monoclonal antibody against H3K9Me3 for 1.5 h at room temperature, and mouse monoclonal antibody against p21^CIP1^ overnight at 4°C. After incubation, slides were then rinsed in PBS before being treated with Super Sensitive polymer—HRP IHC Detection System (DQ400-60KE, BioGenex) followed by incubation with secondary antibody for 45 min at room temperature. Slides were then rinsed in PBS and incubated with polymer—horseradish peroxidase (HRP) reagent for 30 min at room temperature and rinsed in PBS.

After that, the substrate solution containing Diaminobenzidine Chromogen (DAB) was used for 10 min and the slides were washed in distilled water. For the staining step, slides were then lightly counterstained with hematoxylin for 3 min and then washed with tap water. After hematoxylin staining, slides were treated with lithium carbonate solution for 30 s and then washed using tap water. Finally, the slides were dehydrated through ascending concentration of ethanol (95, 100%) and rinsed in xylene. The slides were mounted using dibutyl phthalate in xylene (DPX).

Each staining series had positive control slides (for Lamin B1: normal colon epithelium, for H3K9Me3: human colon carcinoma, for p21^CIP1^: human bladder carcinoma) and the negative control slides. Negative controls were performed by omitting the specific primary antibody (replaced by PBS) from the staining procedure on the same tissue samples that were utilized as positive controls.

### Antibodies and expression evaluation

All antibodies were stored at either 4°C or −20°C as per the manufacturer’s instructions. Antibodies were diluted to the required concentrations for staining of sections for all experiments in PBS. The primary antibodies used were a monoclonal antibody raised against Lamin B1 (catalogue number NBP2-59783, 1:250 dilution; Novus Biologicals, CO, U.S.A.), a monoclonal antibody for H3K9Me3 (clone 6F12-H4, 1:200 dilution; Novus Biologicals, CO, U.S.A.), and a monoclonal antibody for p21 (WA-1 (HJ21), 1:50 dilution; Novus Biologicals, CO, U.S.A.). A polymer—HRP reagents conjugated to anti-mouse and anti-rabbit secondary antibody (DQ400-60KE; BioGenex, U.S.A.) were used.

Nuclear staining of Lamin B1, H3K9Me3, and p21^CIP1^ was scored semi-quantitatively in the most prominently stained area of the tissue slides measuring stained cells and/or area ratio by two independent pathologists using a light microscope (Olympus BX 25, Olympus, Tokyo, Japan) under 20× and 40× objective lenses. The expression assessment of the Lamin B1, H3K9Me3, and p21^CIP1^ markers and the cutoffs used were based on previous studies [33–35]. For Lamin B1, any nuclear staining in the tumor samples was considered positive, while tumor samples with <10% positively stained tissue were considered negative. For H3K9Me3 and p21^CIP1^, tumor samples with >50% positively stained tissue were considered positive while tumor samples with ≤50% positively stained tissue were considered negative.

Determining senescence induction was based on the evaluation of all three tested biomarkers combined. In that, only samples that show positive expression for H3K9Me3 and p21^CIP1^ and negative expression of Lamin B1 were considered positive for senescence. Consequently, samples that were negative for H3K9Me3 and p21^CIP1^ and positive expression of Lamin B1 were considered negative for senescence. Lastly, all other expression possibilities were considered as inconclusive for senescence.

### Patient follow-up

Using patient databases of the Departments of Surgery at JRMS and PHH, postoperative patient follow-up status was evaluated. All patients (*n*=55) were followed-up for evidence of secondary disease (i.e., recurrence) until 1 December 2020 (median follow-up period, 18 months following the time of operation). Evidence of cancer recurrence/metastasis following therapy was also confirmed through the Oncology Department at JRMS and Department of Surgery at PHH. Breast cancer recurrence/metastasis was confirmed using clinical assessment, ultrasonography, mammography, computed tomography (CT), Magnetic Resonance Imaging (MRI), or Whole-body Positron emission tomography (PET) scans.

### Statistical analysis

The relationship among Lamin B1, p21^CIP1^, and H3K9Me3 expression were derived using Fisher’s exact test. Correlations among the different variables were computed using the Chi-square (χ^2^) test and Fisher’s exact test. Fisher’s exact test was used for dataset comparisons of *n*<5. Wilcoxon signed-rank and McNemar’s tests were used to evaluate the difference between the pre-and post-NAC samples. The results were considered as statistically significant with *P*-values <0.05, whereas *P*-values ≥0.05 were taken as non-significant. Data analysis was performed using IBM SPSS Statistics Version 24.

## Results

### Evaluation of breast tumors based on response to NAC

Our initial analysis identified a total number of 89 breast cancer patients who were diagnosed with breast cancer and receiving one form of NAC in two centers. Of those, ten patients were deceased while receiving surgical or medical treatment and were excluded from the analysis (**Supplementary Figure S1**). All patients were females with an average age of 48.65 years (Table 1). All patients’ disease was confirmed as invasive breast carcinoma through core needle biopsy prior to receiving any form of therapy and approx. 92.4% of the patient samples were diagnosed with IDC, while only 7.6% were diagnosed with ILC of stages I–III (Table 1). Most tumors were graded as G2 (65.8%) (Table 1). The receptor status of all 79 tumors was determined through core needle biopsy prior to onset of therapy. Of those, 64.6% were hormone receptor positive (Estrogen Receptor positive, ER+, and Progesterone Receptor positive, PR+), 29.1% were Human Epidermal Growth Factor Receptor 2 positive (HER2+) and 13.9% had triple-negative disease (Table 1).

**Table 1:** Clinical and pathological characteristics of patients’ sample

Clinical and pathological characteristics	Number of patients (%)
**Patients age (years)**	
<50	42 (53.2%)
≥50	37 (46.8%)
**Histopathological tpe**	
Ductal	73 (92.4%)
Lobular	6 (7.6%)
**Grade**	
G1	4 (5.1%)
G2	52 (65.8%)
G3	23 (29.1%)
**ER status**	
Negative	20 (25.3%)
Positive	59 (74.7%)
**PR status**	
Negative	25 (31.6%)
Positive	54 (68.4%)
**HER2 status**	
Negative	56 (70.9%)
Positive	23 (29.1%)
**Pathologic LN status**	
Negative	27 (34.2%)
Positive	48 (60.7%)
Unknown	4 (5.1%)

Table shows description of patient data regarding age, histopathological breast cancer type, grade, hormone receptor expression status including ER and PR, and HER2, and lastly, pathological lymph node (LN) status. All patients (*n*=79) received a form of neoadjuvant therapy prior to undergoing radical mastectomy. Of those, 55 patients received NAC only (*n*=55), while 24 patients were concomitantly treated with radiation and/or hormonal (e.g., tamoxifen) therapies prior to surgical resection (*n*=24). The age of patients ranged from 31 to 71 years, with a mean of 48.7 years.

Of the remaining 79 patients, 55 patients received NAC only, while 24 patients were concomitantly treated with radiation and/or hormonal (e.g., tamoxifen) therapies prior to surgical resection and were excluded from the biochemical staining (**Supplementary Figure S1**). The percentage of patients that developed complete pathologic response (pCR, ypT0/is, ypN0) to NAC combined with either radiation or hormonal therapies was 18.9%, while the remaining 81.1% had partial or incomplete pathological response to therapy. In comparison, the percentage of patients who developed pCR to NAC alone was 27.3%, while the remaining 72.7% had partial or incomplete pathologic response to sole NAC. Interestingly, 33.3% of patients who developed pCR following NAC were positive for ER and PR and 40.0% were negative for HER2 receptor (Table 2). On the contrary, 67.5% of patients who developed partial or incomplete response to NAC were positive for ER and PR and 27.5% were positive for HER2 receptor (Table 2). These results indicate that, in consistence with previous reports [36,37], breast tumors exhibiting positive expression for hormone receptors (both ER and PR) and negative expression for HER2 receptor have significantly poor response to NAC (*P*=0.022 and *P*=0.026, respectively, Table 2).

**Table 2:** Association of hormone receptor status with the pathological response following NAC

	Number of patients (%)	Number of patients with pCR (%)	Number of patients with partial or incomplete response (%)	
**ER and PR**				
Positive	32 (58.2%)	5 (33.3%)	27 (67. 5%)	*P=*0.022*
Negative	23 (41.8%)	10 (66.7%)	13 (32.5%)	
**HER2**				
Positive	20 (36.4%)	9 (60%)	11 (27.5%)	*P=*0.026*
Negative	35 (63.6%)	6 (40%)	29 (72.5%)	

Table describes the correlation between the breast cancer samples with evidence of pCR or partial or incomplete pathological response in all patients that received NAC only (*n*=55) to hormone receptor status (ER, PR) and HER2. Data indicate that breast tumors exhibiting positive expression for ER and PR and negative expression for HER2 receptor have significantly poor response to NAC. * indicates statistical significance based on *P*-values as calculated using χ^2^ test.

Bold values indicate* P*<0.05.

### Expression of senescence markers H3K9Me3, p21^CIP1^, Lamin B1 in breast tumor samples with poor or incomplete pathological response to NAC

To examine if TIS is a component of the incomplete clinical response to therapy, we investigated the expression of TIS-associated protein markers only in tumor samples of patients who received NAC prior to surgery but did not develop pCR (*n*=37). The clinical information regarding age, tumors, stage, tumor grade, receptor status type, and type of NAC of individual patients are provided in **Supplementary** **Table S1**. The investigated biomarkers include p21^CIP1^, a CDKI involved in the regulation of the senescent growth arrest [38], H3K9Me3, a component of the SAHF [39], and Lamin B1, the loss of which is reflective of nuclear envelope remodeling associated with senescence induction [40].

**Figure 1:**
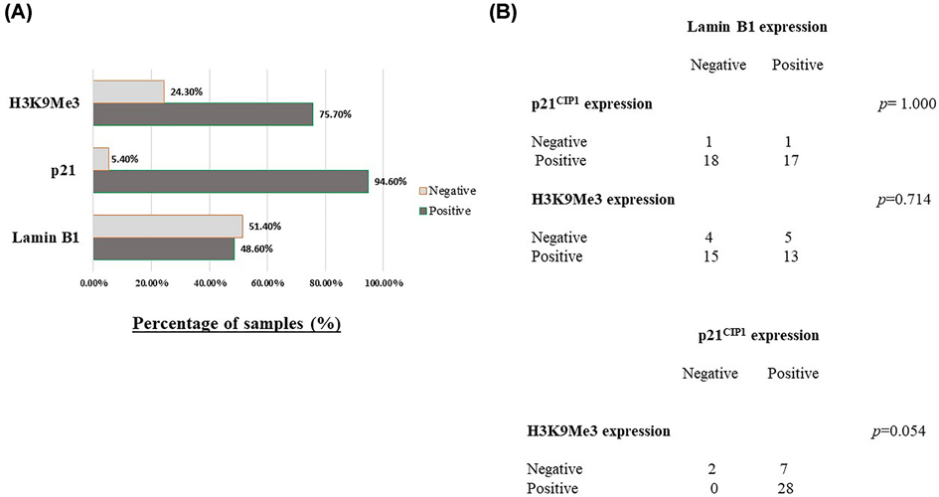
Expression levels of p21^CIP1^, H3K9Me3, and Lamin B1 in breast cancer samples following NAC (**A**) The percentage of samples with positive or negative expression of each senescence-associated biomarker in samples of patients that only received NAC and developed partial or incomplete pathological response to therapy (*n*=37). (**B**) Table demonstrates correlational expression of the three tested senescence-associated biomarkers. Upper panel shows direct comparison of Lamin B1 expression with both p21^CIP1^ and H3K9Me3 separately, while lower panel shows direct comparison of p21^CIP1^ expression with H3K9Me3 expression. *P*-values are included as calculated using Fisher’s exact test.

The expression of p21^CIP1^, H3K9Me3, and Lamin B1 was immunohistochemically determined in paraffin-embedded tumor samples obtained intraoperatively following the completion of NAC treatment and scoring for these markers was performed as described previously [33–35]. Immunoreactivity of p21^CIP1^, H3K9Me3, Lamin B1 was localized in the nuclei of tumor cells in consistence with their typical expression (**Supplementary** **Figure S2**). Our analysis showed that the expression of p21^CIP1^, H3K9Me3, and Lamin B1 in tumor samples of patients who developed partial or incomplete response to NAC was 94.6, 75.7, and 48.6%, respectively (Figure 1A). There was no correlation among Lamin B1, H3K9Me3, and p21^CIP1^ when their individual expressions where compared (Fisher’s exact test, *P*=0.714, *P*=1.000, and *P*=0.054) (Figure 1B). For example, samples that were positive for p21^CIP1^ or H3K9Me3 were not necessarily negative for Lamin B1 (Figure 1B). Furthermore, we found no significant relationship between the expressions of Lamin B1, H3K9Me3, p21^CIP1^, and clinicopathologic parameters including the stage of the tumor, histologic tumor grade, and hormone receptor status in patients who received NAC (Table 3). However, expression level of Lamin B1 is significantly correlated with tumor grade (Table 3). To our knowledge, there is limited investigation of the H3K9Me3 and Lamin B1 expression in human breast cancer and this is the first study to indicate the levels of H3K9Me3 expression and Lamin B1 expression in breast cancer samples following exposure to NAC.

**Table 3:** Correlation among p21^CIP1^, H3K9Me3, and Lamin B1 expression with clinicopathological variables

	p21^CIP1^		H3K9Me3		Lamin B1	
	-	+	-	+	-	+
**Grade**	*P* **=0.922**		*P*=**0.677**		*P*=0.032*	
G1	0	2	0	2	2	0
G2	1	19	5	16	7	14
G3	1	14	4	10	10	4
**Stage**	*P*=**0.565**		*P*=**0.564**		*P*=**0.092**	
I	0	3	0	3	0	4
II	0	10	3	7	6	4
III	2	22	6	18	13	10
**ER status**	*P* **=1.000**		*P**=*** **1.000**		*P* **=1.000**	
Positive	2	27	7	23	15	14
Negative	0	8	2	6	4	4
**PR status**	*P* **=1.000**		*P* **=0.679**		*P=* **1.000**	
Positive	2	25	6	21	14	13
Negative	0	10	3	7	5	4
**HER2 status**	*P* **=1.000**		*P* **=0.159**		*P* **=0.693**	
Positive	0	8	0	8	5	3
Negative	2	27	9	20	14	15

Table describes statistical correlation of the expression status of each senescence-associated biomarker and several clinicopathological variables including grade (G), stage, receptor expression (ER, PR, HER2). There was no significant correlation between the expression of the three tested biomarkers with any of the analyzed clinicopathological variables with the exception of Lamin B1, which was significantly correlated with tumor grade. * indicates statistical significance based on *P*-values as calculated using χ^2^. Both χ^2^ and Fisher’s exact tests were utilized in data analysis.

Bold values indicate* P*<0.05.

**Figure 2:**
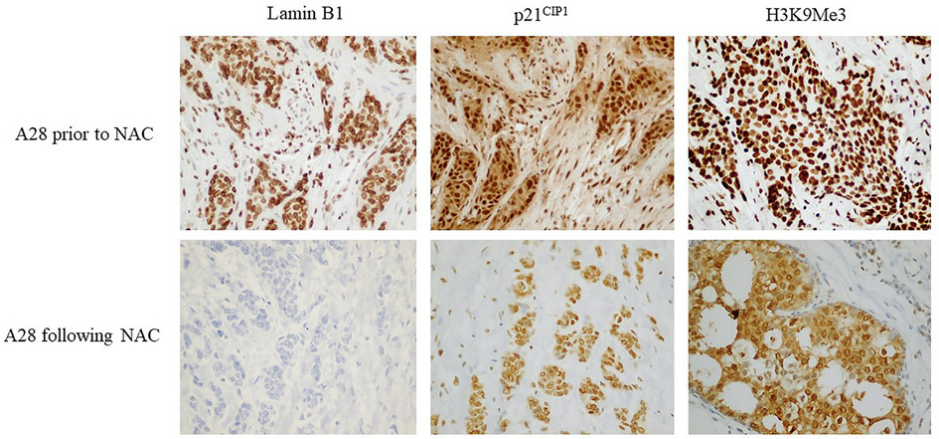
Expression levels of p21^CIP1^, H3K9Me3, and Lamin B1 in breast cancer samples before and after NAC Pre-NAC staining was performed on core needle biopsy samples of 11 patients that received NAC only and developed partial or incomplete pathological response (*n*=11). Representative bright-field microscopic images of the immunohistochemical expression levels of p21^CIP1^, H3K9Me3, and Lamin B1 in breast cancer sample of patient A28, showing insignificant reduction in p21^CIP1^ and H3K9Me3 expression levels, but significant decrease in Lamin B1 levels following NAC. All images were taken using bright-field microscopy (Olympus BX 25, Olympus, Tokyo, Japan) under 40× objective lens.

To further investigate if the changes in expression of the three TIS-associated markers were due to exposure to NAC, immunohistochemical staining of Lamin B1, p21^CIP1^, and H3K9Me3 was performed on available core-needle biopsy samples (*n*=11) which were collected prior to receiving NAC (Figure 2). Interestingly, and to our surprise, we observed high expression levels of p21^CIP1^ and H3K9Me3 in the core needle biopsy samples prior to the exposure of breast tissue to NAC, and a decrease in the expression level of p21^CIP1^ and H3K9Me3, albeit non-significant, following NAC (*P*=0.109/*P*=0.500, *P*=0.114/*P*=0.250; based on Wilcoxon’s and McNemar’s tests, respectively) (Table 4). However, we found that there are significant changes in the expression rate of Lamin B1 pre- and post-chemotherapy (*P*=0.003/*P*=0.031; based on Wilcoxon’s and McNemar’s tests), where the expression rate of Lamin B1 decreased from 100% to 45.5% following receiving NAC (*P=*0.003/*P*=0.031; based on Wilcoxon’s and McNemar’s tests), which is more consistent with the development of TIS (Table 4). In order to eliminate the possibility of a staining artifact, which could have accounted for the high expression level of p21^CIP1^ and H3K9Me3 in the biopsy specimens, we stained three breast tumor samples from patients who only underwent mastectomy without prior exposure to NAC and found high expression levels of both markers (**Supplementary** **Figure S3**).

**Table 4:** Immunohistochemical expression levels of p21^CIP1^, H3K9Me3, and Lamin B1 before and after NAC

	Number of positively stained pre-NAC samples (%)	Number of positively stained post-NAC samples (%)	Wilcoxon’s test	McNemar’s test
**p21^CIP1^**	11 (100%)	9 (81.8%)	*P*=**0.109**	*P*=**0.500**
**H3K9Me3**	11 (100%)	7 (63.6%)	*P*=**0.114**	*P=* **0.250**
**Lamin B1**	11 (100%)	5 (45.5%)	*P*=0.003*	*P=* 0.031*

Of analyzed 37 breast tumor samples, 11 patients had available core needle biopsy samples (*n*=11) of breast tissue prior to receiving NAC. Numbers and percentages reflect positive staining of each analyzed biomarker prior to NAC (in core needle biopsy samples) and following NAC. Both p21^CIP1^ and H3K9Me3 did not exhibit significant changes in expression prior and post receiving NAC. Lamin B1 expression was significantly decreased following NAC. *P-values recorded are the results from Wilcoxon’s signed-rank and McNemar’s tests.

Bold values indicate* P*<0.05.

**Figure 3:**
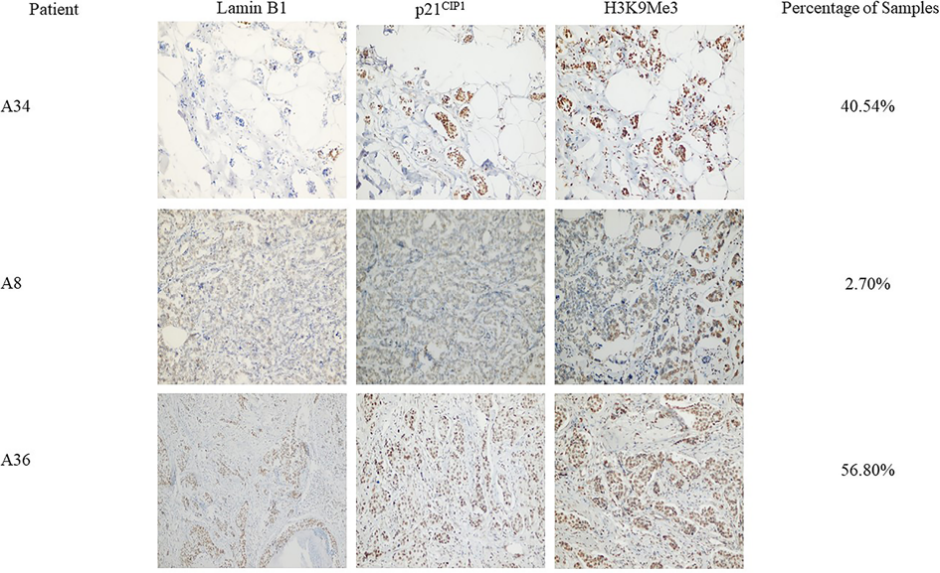
Determination of evidence on the induction senescence-like phenotype based on p21^CIP1^, H3K9Me3, and Lamin B1 staining Determining senescence induction was based on the evaluation of all three tested biomarkers combined. Only samples that show positive expression for H3K9Me3 and p21^CIP1^ and negative expression of Lamin B1 were considered positive for senescence. Consequently, samples that were negative for H3K9Me3 and p21^CIP1^ and positive expression of Lamin B1 were considered negative for senescence. Lastly, all other expression possibilities were considered as inconclusive for senescence. Upper panel shows representative images for patient A34 whose samples were positive for p21^CIP1^, H3K9Me3 and negative for Lamin B1, and hence, considered positive for senescence-like phenotype. Conversely, middle panel shows representative images for patient A8 whose samples were negative for p21^CIP1^, H3K9Me3 and positive for Lamin B1, and hence, considered negative for senescence-like phenotype. Other possibilities of expression, as shown in representative images in lower panel (patient A36) were considered inconclusive for senescence-like phenotype. The percentage of samples positive for senescence-like phenotype was 40.54% while the remainder were either negative or inconclusive (2.70 and 56.8%, respectively) (*n*=37). All images were taken using bright-field microscopy (Olympus BX 25, Olympus, Tokyo, Japan) under 40× objective lens.

However, in order to assess senescence induction in breast tumor samples based on the expression levels of the three markers, we considered samples that are only positive for p21^CIP1^, positive for H3K9Me3 and negative for Lamin B1 are most likely to be positive for a senescence-like phenotype post-NAC in consistence with the premise of utilizing multiple senescence-associated markers when evaluating senescence *in vivo* [27]. All the other possibilities were considered either negative for senescence (negative for p21^CIP1^, negative for H3K9Me3, and positive for Lamin B1) or inconclusive (variable marker expression pattern). Accordingly, 15 samples were positive for senescence (40.54%), while the remainders were either negative or inconclusive for senescence (2.70 and 56.8%, respectively) (Figure 3). In addition, based on the same criteria for determining samples positive for senescence-like phenotype (positive for p21^CIP1^ and H3K9Me3 and negative for Lamin B1) none of the 11 core-needle biopsy samples collected prior to receiving NAC were positive (0%, *n*=11). Lastly, we wanted to investigate a possible connection between hormone receptor status of patients who received NAC and senescence-like phenotype induction. Interestingly, most samples that were positive for a senescence-like phenotype based on the expression status of the previous 3 markers were also ER-positive (80%) or PR-positive (73.3%). The percentage of hormone receptor-positive samples that were also positive for senescence-like phenotype was 66.7% (Table 5). Interestingly, only 77.3% of senescence-negative or -inconclusive samples were ER-positive, 77.3% were PR-positive and 72.7% were positive for both receptors.

**Table 5:** Correlation of senescence-like phenotype status and hormone receptor status

	Number of positive for senescence-like phenotype (%)	Number of negative or inconclusive for senescence (%)	
**ER status**			
Positive	12 (80%)	17 (77.3%)	*P=***1.000**
Negative	3 (20%)	5 (22.7%)	
**PR status**			
Positive	11 (73.3%)	17 (77.3%)	*P=***1.000**
Negative	4 (26.7%)	5 (22.7%)	
**ER and PR status**			
Positive	10 (66.7%)	16 (72.7%)	*P=***0.728**
Negative	5 (33.3%)	6 (27.3%)	
**HER2**			
Positive	5 (33.3%)	3 (13.6%)	*P=***0.228**
Negative	10 (66.7%)	19 (86.4%)	

Table demonstrates a correlation between the receptor expression status (ER, PR, HER2) and evidence of positivity for the TIS-like phenotype versus no or inconclusive evidence for senescence-like phenotype. Samples were considered positive for senescence-like phenotype based on the concomitant positive expression of p21^CIP1^ and H3K9Me3 and negative expression of Lamin B1. Other possibilities were considered negative or inconclusive for senescence. * indicates statistical significance based on *P*-values as calculated using Fisher’s test.

Bold values indicate* P*<0.05.

### The connection between breast cancer recurrence and pathological response to NAC, hormone receptor status, and the expression of senescence-associated biomarkers

Next, we wanted to examine whether there is a connection between the induction of a senescence-like phenotype in patients who received NAC and did not develop pCR and the development of secondary metastasis (local or distant recurrent breast cancer disease) following NAC and radical mastectomy. The number of patients that confirmed evidence of local or distant recurrence following surgical resection was 8 (14.5%) (Table 6). As expected, the number of patients who had a pCR to NAC had a lower incidence of developing recurrent disease in comparison to patients whose tumors poorly responded to NAC (Table 6). Moreover, up to 10.9% of patients developed recurrent disease and were also positive for hormone receptors (ER and PR), while 3.6% of patients developed recurrent disease and were HER2-positive (Table 6). Lastly, it was evident that of the eight patients with partial or incomplete response to NAC that developed recurrent metastatic disease, three tumor samples were positive for senescence-like phenotype. The median patient follow-up duration was 18 months postoperatively, indicating that our analysis investigated only short-term recurrence levels. These results do not establish a strong connection between TIS precipitated in breast tumor cells by NAC and the incidence of short-term breast cancer recurrence over a period of 18 months postoperatively.

**Table 6:** Correlation of cancer recurrence with pathologic response, hormone receptors status, and senescence-like phenotype

	Evidence of local or distant recurrence (%)	No evidence of local or distant recurrence (%)	
**pCR**	**0 (0%)**	**15 (27.3%)**	*n*=55, *P*=**0.091**
**No pCR**	**8 (14.5%)**	**32 (58.2%)**	
**ER/PR (+)**	**6 (10.9%)**	**26 (47.3%)**	*n=*55, *P*=**0.446**
**ER/PR (−)**	**2 (3.6%)**	**21 (38.2%)**	
**HER (+)**	**2 (3.6%)**	**18 (32.7%)**	*n=*55, *P*=**0.257**
**HER2 (−)**	**6 (10.9%)**	**29 (52.7%)**	
**Senescence (+)**	**3 (8.1%)**	**12 (32.4%)**	*n=*37, *P*=**1.000**
**Senescence (−)**	**5 (13.5%)**	**17 (45.9%)**	

Table shows the numbers and percentages of patients who developed local or distant cancer recurrence based on the following variables: pathological response to NAC, hormone receptor status (ER, PR), HER2 status, and senescence-like phenotype status. Patients who developed pCR did not develop local or distant metastasis over the follow up period of 18 months. No significant correlation between recurrence and other variables was recorded, including in patients’ samples positive for senescence-like phenotype. *P-*values were calculated using Fisher’s exact test.

Bold values indicate* P*<0.05.

## Discussion

NAC has been shown to improve the outcome in patients with locally advanced breast cancer and is considered a standard of care for HER2-positive or triple-negative breast cancer [41–43]. NAC can contribute to the reduction in the tumor size prior to surgery which increases the chance of successful resection and might reduce the rate of recurrence and distant metastasis [44,45]. However, some data indicate that approximately 10–35% of patients do not benefit from this clinical treatment approach [46–48]. For example, in the NSABP B-27 neoadjuvant trials, locoregional recurrence rate at 10 years for breast cancer patients who received TAC neoadjuvant regimen was 9.5% [49]. In addition, a recent report indicated that the locoregional recurrence rate in breast cancer patients was 21.4% within 15 years after NAC treatment [8]. In contrast, the National Surgical Adjuvant Breast and Bowel Project (NSABP) and the European Organization Research and Treatment of Cancer (EORTC) conducted studies related to the comparison of neoadjuvant versus adjuvant systemic therapy in women with operable breast cancer [50], and concluded that there was no difference in overall survival, progression-free survival, or time to local-regional recurrence following preoperative chemotherapy [50].

Accordingly, local and distant recurrence of breast cancer following anticancer therapy remains to be a major challenge to successful, eradicating cancer treatment. Unfortunately, the underlying molecular and pharmacological mechanisms of tumor dormancy followed by cancer recurrence are not fully elucidated. Interestingly, the recurrence rate was higher in certain breast cancer populations receiving anthracycline-based NAC compared with patients that did not receive the same treatment, highly suggesting the potential association between cellular responses to chemotherapy and cancer relapse [8].

Molecularly, responses of tumor cells to cancer therapy include apoptosis, necroptosis, autophagy, mitotic catastrophe, and several forms of growth arrest such as cellular senescence [51]. While the ideal response to therapy is apoptosis, evidence has shown that it is not always the case, and cells can alternatively undergo senescence [52–55]. Senescence has traditionally been defined as an ‘irreversible’ form of growth arrest, and the characteristic cytostatic nature of senescence encouraged the utilization of senescence-inducing therapy (traditional DNA damaging chemotherapy) [56]. However, several reports recently have confirmed that TIS in tumor cells is not obligatorily irreversible and that some tumor cells can escape the stable senescent growth arrest (reviewed in [16]). Moreover, senescent tumor cells have been shown to acquire stem-cell like characteristics [57], leading to more aggressive phenotypes [58], and can contribute to adverse outcomes of cancer therapy [59], which led to the proposal of TIS as a mechanism of tumor dormancy and cancer recurrence [20]. Accordingly, our analysis aimed to investigate the induction of TIS in breast cancer samples that underwent partial or incomplete pathological response to NAC.

Several markers are commonly used to identify senescence activation *in vitro* [60]. However, senescence-associated markers independently are not specific to assay establishment of senescence both under *in vitro* and *in vivo* conditions until now [61]. Accordingly, identifying senescence *in vivo* should be based on the use of multiple senescence-associated markers [27]. Numerous *in vitro* studies showed that increased expression of p21^CIP1^, H3K9Me3, and reduced expression of Lamin B1 as evidence of senescence induction [26,28,30,62,63]. In our study, we investigated the expression of p21^CIP1^, H3K9Me3, and Lamin B1 using immunohistochemistry (IHC) to evaluate TIS induction in breast cancer patients who received NAC and developed partial or poor response to therapy.

In the current work, we observed a slight, non-significant decrease in the expression levels of p21^CIP1^, and H3K9Me3 (although both were positively expressed in most samples) and a significant decline in the expression of Lamin B1 in patients who received NAC and that, based on staining for all three markers, senescence-like phenotype was identified within advanced breast cancers in response to NAC. Up-regulation of p21^CIP1^ protein leads to cell cycle arrest by suppressing transition from G_1_ phase into S-phase [64], and is an established feature of senescence in different breast cancer cell lines *in vitro* [65,66]. In the present study, we found that expression of p21^CIP1^ expression in breast cancer tissues after NAC was high, despite no significant increase in p21^CIP1^ expression levels following NAC, based on the lack of a significant change in expression levels comparing pre- and post-NAC immunohistochemical staining. Moreover, statistical analysis suggested that high expression of p21^CIP1^ is not significantly correlated with pathologic TNM stage, tumor grade and status of ER, PR or HER2 in patients who received NAC. These findings are in line with a previous study showing a significant reduction in p21^CIP1^ protein expression levels in invasive breast cancer who received NAC regimens (docetaxel in combination with epirubicin) compared with the core biopsy specimens from same breast cancer patients before NAC [67]. In contrast, another study showed an increased expression of p21^CIP1^ in breast cancer in response to NAC [62]. These previous observations, and our results, suggest that p21^CIP1^ might not be a useful senescence-associated marker in the evaluation of TIS in invasive breast cancer due to its variable expression following exposure to DNA damaging NAC.

Similarly, we found that high nuclear expression of H3K9Me3 in patients who received NAC and developed a partial response to therapy (75.7%). A Previous report showed that there is H3K9Me3 positive expression in 71.8% in human breast cancer [34]. Moreover, our statistical analysis suggested that expression of H3K9Me3 is not significantly correlated with pathologic TNM stage, tumor grade and status of ER, PR and HER2 in patients who received NAC in a similar fashion to our observations on p21^CIP1^. The expression levels of H3K9Me3 also have not significantly changed between samples obtained prior to NAC (by core-needle biopsy) or following NAC (as resected during radical mastectomy). Again, these observations indicate that changes in H3K9Me3 expression levels might not be critical in identifying the induction of TIS in breast cancer samples in response to NAC.

On the other hand, our analysis suggested that expression level of Lamin B1 is significantly correlated with tumor grade, but not significantly correlated with pathologic TNM stage or receptor status. Moreover, we observed a significant decline in Lamin B1 expression in patients who received NAC consistent to changes in expression that occurs in *in vitro* senescence [28,40]. Some reports indicated that Lamin B1 expression is reduced in normal human fibroblast and mouse cell lines by various stimuli such as DNA damaging agents, replicative exhaustion, or oncogenic signaling [68]; however, unfortunately, the evidence in clinical breast cancer samples is scarce. Its noteworthy that loss of Lamin B1 expression was identified in different malignant tissue such as prostate, breast and esophageal carcinoma [69], indicating that the decline of Lamin B1 expression might be considered a malignancy biomarker. Overall, our data suggest that Lamin B1 displays a significant change in expression following exposure to NAC and can be considered as a marker for future testing batteries utilized to identify TIS *in vivo*.

Our observations indicate that based on the concomitant expression of three senescence-associated markers, senescent-like phenotypes exist within human breast cancers who received NAC (40.54%). Previous reports suggest that senescence can indeed be induced *in vivo* in response to CAF regimen (cyclophosphamide, doxorubicin, and 5-fluorouracil) based on SA-β-gal, p53 and p16^INK4a^, and showed that 41% of tumors stained positive for SA-β-gal marker while patients with high nuclear p16^INK4a^ (but low nuclear p53 expression) [17]. Moreover, a previous study has shown that the TIS marked by IHC staining for Ki-67, plasminogen activator inhibitor-1, and SA-β-gal was detected in 30 samples of colorectal cancer [70]. These studies should be combined with our data in order to develop better predictive models for the identification of TIS *in vivo*.

Finally, several previous studies reported that the expression of senescence biomarkers correlated with poor outcomes in different kinds of cancers such as breast, colon, and bone cancer [35,71–73]. In addition to determining the extent of senescence induction *in vivo*, the exact role of senescence in determining the outcome of therapy is still debatable [74]. Accordingly, there is uncertainty to the contribution of senescence to the effectiveness of therapy. In that, although chemotherapy-induced senescence has been studied for decades, the impact of senescence on disease control remains uncertain. Senescence can be perceived as a favorable outcome of cancer treatment due to the fact that senescent tumor cells are in a growth-abrogated phase that halts tumor proliferation [75]. On the other hand, the fact that senescent cells are resistant to apoptosis can be interpreted to indicate that senescence-mediated growth arrest serves as a strategy for tumor cells to evade the cytotoxicity of chemotherapy and radiation. The extensive genetic heterogeneity of tumor cells, and specifically, of a senescent tumor population, would, however, suggest that there are subpopulations of these metabolically viable cells that would not obligatorily persist in a permanent senescent-like state of growth arrest [76]. This largely invites for the development of better testing approaches to identify senescent tumor cells *in vivo*.

A limitation to our study is that the number of biomarkers tested is low and the association of their *in vitro* expression does not necessarily correlate with *in vivo* senescence. A better approach shall implement wide-transcriptomic analysis of senescence-associated pathways in order to identify an *in vivo* signature. Such readily available biomarkers can be implemented in carefully investigating the contribution of TIS to therapy outcomes, recurrence rates, and the validity of the recent proposition of using *senolytic* therapy as adjuvant cancer treatment [77]. Senolytic agents are drugs that selectively kill senescent cells, that have been shown to exert remarkable potential to clear senescent cells *in vitro* and *in vivo* in preclinical studies of aging-related pathologies and TIS in tumor models [78–81]. Despite the promising proposal of using senolytics in cancer therapy, a better understanding of *in* *vivo* senescence is critically required. Another limitation to our study is the low number of breast cancer samples collected and the short-term follow up period of patients following the completion of therapy. Large-scale, prolonged studies aimed at the identification of senescent tumor cells and their contribution to clinical cancer outcomes are needed.

## Supplementary Material

Supplementary Figures S1-S3 Table S1

## Data Availability

All data included in the present study are available upon request by contact with the corresponding author.

## Abbreviations

CDKI: cyclin-dependent kinase inhibitor
ER: estrogen receptor
FFPE: formalin-fixed paraffin-embedded
HER2: human epidermal growth factor receptor 2 positive
HRP: horseradish peroxidase
H3K9Me3: histone H3 lysine 9 trimethylation
IDC: invasive ductal carcinoma
IHC: immunohistochemistry
ILC: invasive lobular carcinoma
JRMS: Jordanian Royal Medical Services
NAC: neoadjuvant chemotherapy
PBS: phosphate buffered saline
pCR: complete pathological response
PHH: Prince Hamza Hospital
PR: progesterone receptor
SAHF: senescence-associated heterochromatic foci
SA-β-gal: senescence-associated β galactosidase
TIS: therapy-induced senescence
