# Supplementary material for: Expression of therapy-induced senescence markers in breast cancer samples upon incomplete response to neoadjuvant chemotherapy
Source: Biosci Rep. 2021 May 20;41(5):BSR20210079. doi: 10.1042/BSR20210079 (PMC8725197; doi:10.1042/BSR20210079)
Supplement: Supplementary Figures S1-S3 Table S1 [file BSR-2021-0079_supp.pdf]

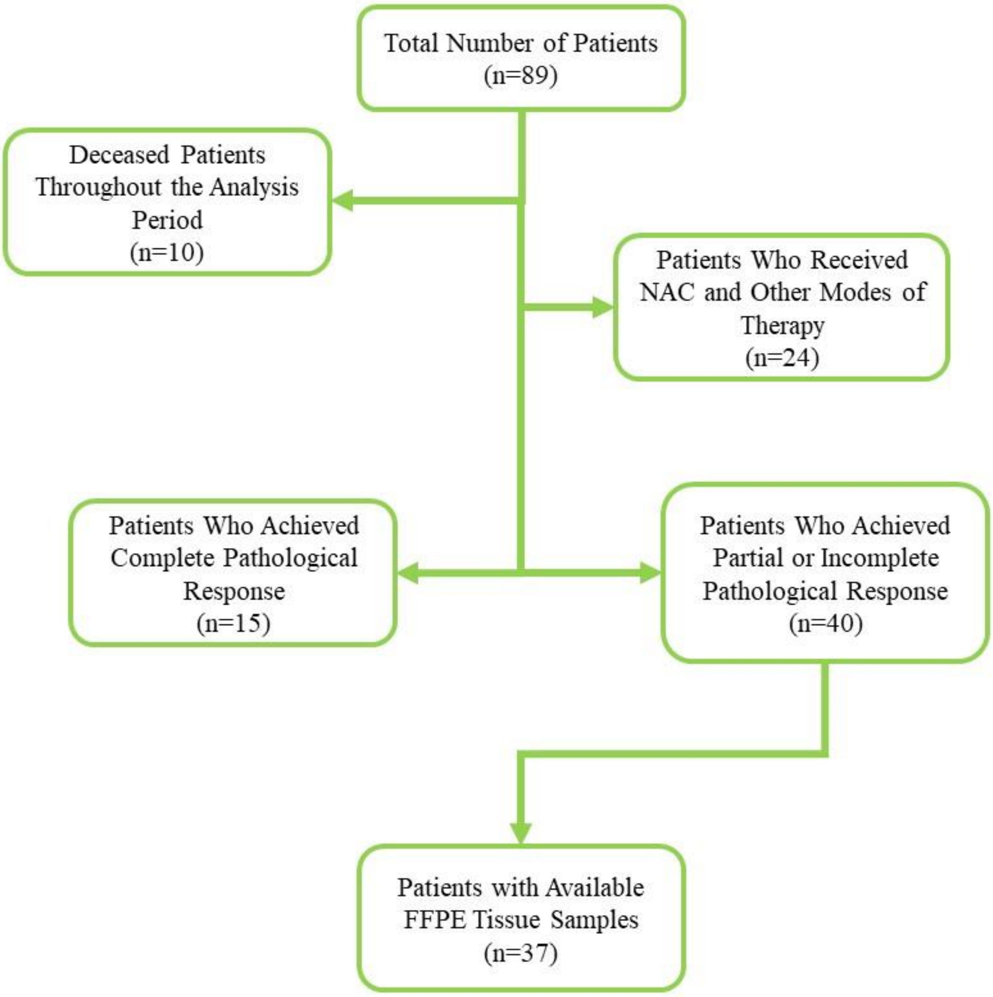

Supplementary Figure 1. **Flow chart depicting the selection process of the tested samples based on the inclusion and exclusion criteria**. A total of 89 patients who were diagnosed with a subtype of breast cancer and received one of the indicated neoadjuvant chemotherapy regimens with variable pathological responses (n=89) were identified in two separate centers. Of those, 10 patients were deceased while receiving treatment and were excluded from the analysis. Furthermore, 24 patients received other treatment modalities including hormonal therapy and/or radiotherapy and thus were excluded from the analysis. Of the remaining 55 patient samples, 15 samples are for patients who developed a complete pathological response, while the remainder (40 patients) developed a partial or incomplete pathological response to neoadjuvant therapy. Finally, a total of 37 patients who had a partial or no response to NAC only had available Formalin-fixed paraffin-embedded (FFPE) samples and were considered for immunohistochemical staining (n=37).

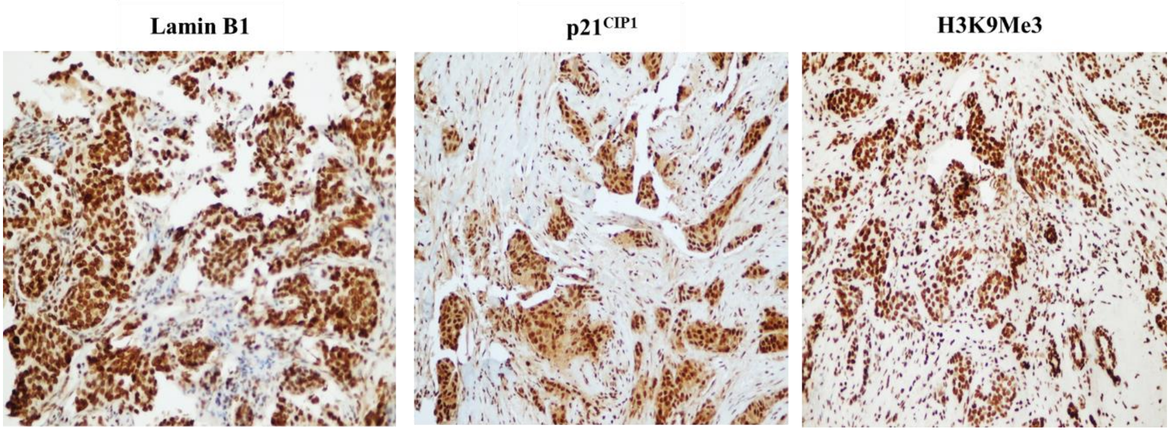

Supplementary Figure 2. The immunohistochemical staining pattern of Lamin B1, p21^{CIP1} and H3K9Me3. Images show nuclear immunohistochemical localization of Lamin B1, p21^{CIP1} and H3K9Me3 in breast tumor samples. All images were taken with a 40X objective.

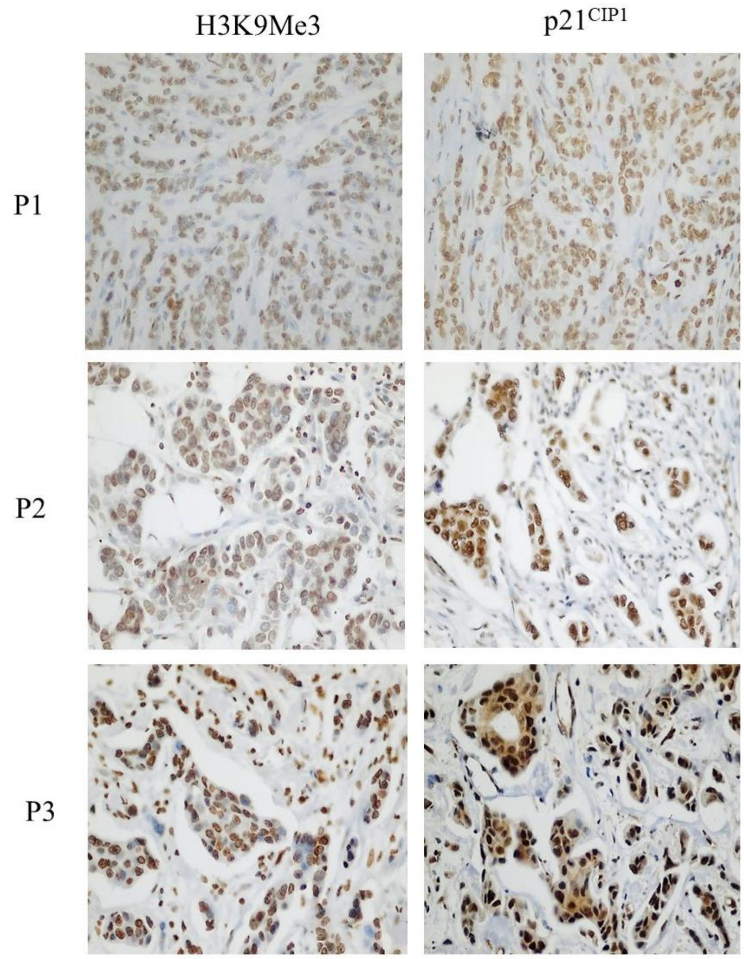

Supplementary Figure 3. Immunostaining of p21CIP1 and H3K9Me3 in breast cancer tissue without exposure to NAC. Images show immunoexpression of $p21^{CIP1}$ and H3K9Me3 markers in breast tumor tissue of three patients who underwent modified radical mastectomy without prior exposure to NAC (n=3). All images were taken with a 40X objective.

Supplementary Table 1. Demographic and histopathologic information and type of chemotherapy received by the studied population. The table delineates patient-specific information including age, type of neoadjuvant chemotherapy received, number of cycles, pathological type of diagnosed breast cancer, grade, Neoadjuvant Pathologic Staging (yp) and hormone receptor status for each individual patient. The average patient age was 50.3 years old. Breast cancer patients received one of the following neoadjuvant chemotherapy regimens: AC, AC+D, FEC or FEC+D, (AC=Adriamycin, cyclophosphamide; D=docetaxel; FEC=5-fluorouracil, epirubicin, cyclophosphamide; T=paclitaxel, IDC=Invasive ductal carcinoma; ILC=Invasive lobular carcinoma, M=mixed, ER=Estrogen Receptor; PR=Progesterone receptor; HER-2=Human epidermal growth factor receptor-2. TNM Staging was based on the 7th edition of American Joint Committee on Cancer Staging System.

Patient	Age	Type and Dose of Neoadjuvant	Histological Tumor Type	Grade	TNM Staging	Receptor Status
**A1**	39	AC*4+ D*4	IDC	G3	pT4bN0	+ER, +PR, -HER2
**A2**	41	AC*4+ D*4	IDC	G3	T2N0	-ER, -PR, -HER2
**A3**	66	AC*4+ D*4	IDC	G3	ypT3 ypN3	+ER, +PR, -HER2
**A4**	71	AC*4+ D*4	ILC	G2	ypT3 ypN3	+ER, +PR, -HER2
**A5**	69	AC*4+ D*4	IDC	G3	ypT2 ypN2	+ER, +PR, -HER2
**A6**	71	AC*4	IDC	G3	ypT4 ypN1b	-ER, -PR, +HER2
**A7**	61	AC*4+ D*4	M (IDC and ILC)	G1	ypT3 ypN3	+ER, +PR, -HER2
**A8**	48	FEC*3+ D*3	IDC	G3	ypT2 N2	+ER, +PR, -HER2
**A9**	53	AC*4+ D*4	IDC	G1	ypT3 ypN2a	+ER, +PR, -HER2
**A10**	46	AC*4+ D*12	ILC	G2	ypT2 ypN3	+ER, +PR, -HER2
**A11**	34	AC*4+ D*11	IDC	G2	ypT2yN2a	+ER, -PR, -HER2

**A12**	35	AC*4+ D*12	IDC	G2	ypT2 ypN0	+ER, -PR, -HER2
**A13**	49	AC*4+ D*4	IDC	G2	N/A	-ER, -PR, -HER2
**A14**	61	AC*4+ D*4	IDC	G2	ypT2 ypN3	+ER, +PR, +HER2
**A15**	70	AC*4+ D*4	IDC	G3	yT4b yN1	+ER, +PR, -HER2
**A16**	31	AC*4+ D* 12	IDC	G3	ypT3 ypN0	-ER, -PR, -HER2
**A17**	50	AC*4 + D*3	IDC	G3	pT4N3	-ER, -PR, -HER2
**A18**	64	AC*4+ D* 12	IDC	G3	ypT2 yN0	+ER, +PR, +HER2
**A19**	37	AC*4+T*12	IDC	G2	mypT4bypNx	+ER, +PR, -HER2
**A20**	53	FEC*3+ D*3	IDC	G3	ypT2 ypN3	+ER, +PR, -HER2
**A21**	52	AC*4+T*12	IDC	G3	ypT3 ypN0	-ER, -PR, -HER2
**A22**	38	AC*4+ D*4	IDC	G3	ympT2N3a	+ER, +PR, +HER2
**A23**	46	AC*4+ D*4	IDC	G2	ypT2 ypN0 Mx	+ER, +PR, -HER2
**A24**	62	AC*4+ D*4	IDC	G2	ypT2 ypN1a ypMx	+ER, +PR, -HER2
**A25**	64	AC*4+ D*4	IDC	G2	ypT2 ypN2	+ER, +PR, -HER2
**A26**	50	FEC*3+ D*3	IDC	G2	mypT4bypN3	+ER, +PR, -HER2
**A27**	42	FEC*3	IDC	G2	ympT2N1a	+ER, +PR, -HER2
**A28**	51	AC*4+ T*12	IDC	G2	ypT3	+ER, +PR, -HER2
**A29**	45	AC*4+ D*4	IDC	G2	pT2N1	+ER, +PR, -HER2
**A30**	48	AC*4+ D*4	IDC	G2	T1cN0M0	+ER, +PR, -HER2
**A31**	56	AC*4+ D*4	IDC	G2	pT2N2aM0	+ER, +PR, +HER2

**A32**	40	AC*4+ D*4	IDC	G2	ypT2, ypN1, ypM1	+ER, +PR, +HER2
**A33**	36	AC*4+ D*4	IDC	G2	ypN0, ypM0	-ER, -PR, -HER2
**A34**	36	AC*4+ D*4	IDC	G3	ypT1ypN2ypM0	+ER, +PR, +HER2
**A35**	55	AC*4+ D*4	IDC	G2	ypT1b, ypN0, ypM0	+ER, +PR, -HER2
**A36**	50	AC*4+ D*4	IDC	G2	pT3N3M1	-ER, -PR, +HER2
**A37**	42	AC*4+ D*4	IDC	G2	ypT1bN1M0	+ER, +PR, -HER2
